# Synergistic associations of depressive symptoms and aging on cognitive decline in early Parkinson’s disease

**DOI:** 10.1016/j.prdoa.2023.100192

**Published:** 2023-03-11

**Authors:** Lea Hemphill, Yenny Valenzuela, Kenya Luna, Sarah M. Szymkowicz, Jacob D. Jones

**Affiliations:** a California State University San Bernardino, San Bernardino, CA, USA; b Department of Psychiatry & Behavioral Sciences, Vanderbilt University Medical Center, Nashville, TN, USA

**Keywords:** Parkinson's disease, Depression, Aging, Cognitive impairment

## Abstract

**Objective:**

Parkinson's disease (PD) is the second most common progressive neurodegenerative disorder. About 40%–50% of PD patients experience depression, making it one of the most common neuropsychiatric disturbances in PD. Cognitive deficits (e.g., difficulties with memory, attention) are an additional common complication in PD. Past studies among healthy aging individuals suggest that depression is a risk factor for cognitive decline, and the risk increases with older age. This study aims to examine the association between depressive symptoms on cognitive decline as a function of age among patients with PD. It is hypothesized that older PD patients with more severe depressive symptoms will be at greater risk of cognitive decline than their younger or less depressed counterparts.

**Methods:**

Four hundred and eighty-seven newly diagnosed patients with PD, were assessed for depression and cognition over a five-year period. Participants completed neuropsychological tests that assessed memory, learning, attention, visuospatial functioning, processing speed, and verbal fluency. Multilevel-modeling was used to examine the longitudinal association between cognition, age, and depressive symptoms.

**Results:**

Our results indicated a significant three-way interaction (age X occasion X depressive symptoms) predicting language and working memory/attention performance. More specifically, detrimental associations of depressive symptoms on cognitive decline in these domains were more pronounced among older adults.

**Conclusions:**

Our findings support that older PD patients with comorbid depressive symptoms experience greater cognitive decline compared to their younger counterparts. Findings suggest that older individuals with PD may be more vulnerable to neurotoxic effects of depression (e.g., neuroinflammation, HPA axis disruption), and better management of depression could potentially reduce cognitive decline and dementia risk.

## Highlights

• Older age and depressive symptoms interacted in associations with cognitive decline.
• Verbal fluency and working memory associated with age and depressive symptoms.
• Older Parkinson’s adults may be more vulnerable to neurotoxic effects of depressive symptoms.
• Better management of depressive symptoms in PD individuals may reduce cognitive decline.

## 1 Introduction

Parkinson’s disease (PD) is the second most common progressive neurodegenerative disorder [1]. PD is mainly recognized by its motor symptoms, such as tremors, stiffness, slowed movement, and writing difficulties. However, a variety of non-motor symptoms are also common and may include depression and cognitive deficits.

Depression may be the most common neuropsychiatric disturbance among individuals with PD; affecting about 40 %–50 % of PD patients [2]. Depression in PD can occur at any motor stage and sometimes can be noted prior to onset of motor symptoms [3]. This increased risk of depression in PD is frequently attributed to greater psychosocial stress/consequences (e.g., loss of employment, role expectations, communication), however, neurobiological factors such as disruption of frontal-subcortical circuits important for emotional processing also play a role [[4], [5]]. Depression among individuals with PD is also associated with reduced quality of life and difficulties in activities of daily living [6].

Cognitive impairment is another common non-motor symptom in PD. Cognitive impairment can include difficulties with learning, remembering, concentrating, planning, attention, and organizing tasks, and these impairments can contribute to disability [[7], [8]]. About 25 % of PD patients are diagnosed with mild cognitive impairment (PD-MCI) during their initial PD diagnosis, with up to 80 % experiencing Parkinson’s disease dementia (PDD) within 15–20 years of diagnosis [[9], [10]].

Multiple studies have shown a relationship between cognitive impairment and depression among individuals with PD. Specifically, depression is most consistently associated with worse performance on tests of immediate [[11], [12], [13]] and delayed memory [[13], [14]]. Individuals with PD-MCI experience more severe depressive symptoms relative to cognitively normal PD patients [15]. This is cause for concern because about 25 %–28 % of healthy older adults with comorbid MCI and depression will go on to develop dementia [16]. Furthermore, risk for PDD is increased with depression, older age, and lower education [17]. Researchers have proposed the possibility of delaying cognitive deficits by addressing mood symptoms such as depression; but, the effects of psychiatric interventions on cognitive functioning are yet to be determined [15]. On the other hand, some studies have failed to find a significant association between depression and cognitive impairment in PD [18] or found depression to be associated with domains other than memory, such as executive functioning [19] or processing speed [13]. These contrasting findings are not well understood, but may suggest potential moderating effects.

Among adults without PD, depression and age are proposed to be separate but interacting risk factors of cognitive impairment [[20], [21], [22]]. Lockwood and colleagues examined cognitive impairments among age groups (20–60 years vs >60 years) of adults with and without major depression [22]. They found performance on tests of executive functioning and processing speed was significantly associated with an age X depression interaction term. Specifically, performance was lower among older adults with depression, relative to their younger or non-depressed counterparts. Other studies have found depression to be a significant marker of cognitive decline or dementia among older adults. According to Zhong et al. [21], late-life depression (depression among individuals >60 years of age) presented a 6.4 times higher risk for a cognitive decline when compared to non-depressed elders. Approximately 40 % of elders diagnosed with geriatric depression experience neuropsychological impairments; most prominently in verbal fluency, psychomotor, visuospatial skills, and episodic memory [20].

Age and depression are documented risk factors for cognitive decline among individuals with PD [[9], [14], [15]]. However, we are not aware of studies investigating the separate and interactive effects of these variables, which could partially clarify the inconsistent relationship between depression and cognitive decline in PD. Therefore, the purpose of the present study is to examine the longitudinal association between depressive symptoms and cognitive decline as a function of age among individuals with PD. It is hypothesized that older PD patients with comorbid depressive symptoms will be at greater risk of cognitive decline relative to their younger and less depressed counterparts.

## 2 Methods

### 2.1 Participants

Participants were retrieved from the Parkinson’s Progression Marker’s Initiative (PPMI) on July 21st, 2021. The PPMI is a worldwide multisite study of individuals newly diagnosed with PD that has been well characterized in past studies (https://www.ppmi-info.org/). Participants were assessed for depression and cognition every year over a five-year period. From the PPMI dataset we queried individuals with PD from the untreated cohort (as opposed to the pathogenetic variant cohort) who completed measures of depression and neuropsychological testing for at least a single visit (n = 487). All participants provided informed consent and approval from the Institutional Review Board (IRB) was obtained at each site.

## 3 Measures

The short form of the Geriatric Depression Scale (GDS-15) was used to assess for the presence of depressive symptoms over the previous week, with higher scores indicating greater depression [23]. The GDS-15 is a 15-item self-reported, yes/no format scale that has been validated for use among individuals with PD [24].

A series of neuropsychological tests were administered to assess working memory/attention (Letter-Number Sequencing; LNS) [25], visuospatial functioning (Judgment of Line Orientation Test; JOLO) [26], processing speed (Symbol Digit Modalities Test; SDMT) [27], language/semantic fluency (Animal Fluency) [28], learning/immediate verbal memory (sum of trials 1–3 on the Hopkins Verbal Learning Test-Revised; HVLT-R) and delayed verbal recall (delayed free recall trial on the HVLT-R) [29]. Additionally, the Montreal Cognitive Assessment (MoCA) was used to measure global cognitive functioning [30].

The Movement Disorder Society Unified Parkinson’s Disease Rating Scale, part 3 motor score (MDS-UPDRS-III) assessed the severity of motor symptoms. The MDS-UPDRS-III is a clinician rated assessment of motor symptoms and higher scores indicate more severe symptoms.

## 4 Statistical analyses

Multilevel modeling using SPSS version 28 was used to examine the independent and interactive effects of depression and age on cognitive performance. Missing data was accounted for with full information maximum-likelihood parameter estimation. The neuropsychological metric was entered as the dependent variable/outcome and a separate model was computed for each neuropsychological domain (processing speed, attention/working memory, verbal fluency, visuospatial functioning, learning/immediate memory, delayed memory, global cognition). This resulted in a total of seven models. Independent variables included the following main effects: motor severity, age at baseline, depressive symptoms, and occasion (e.g. baseline, year 1… year 5). The 2-way and 3-way interactive effects of age, depressive symptoms and occasion were also entered as independent variables: age X depressive symptoms, age X occasion, depressive symptoms X occasion, and age X depressive symptoms X occasion.

## 5 Results

### 5.1 Demographics and clinical characteristics

Demographic and clinical characteristics are presented in Table 1. Data was available on 487 participants at baseline, 424 at year 1, 421 at year 2, 364 at year 3, 347 at year 4 and 306 in the final year. On average, participants completed 5.01 assessments out of 6 possible assessments (median = 6.0).

**Table 1:** Baseline Demographic and Clinical Characteristics.

(N = 487)	Mean	SD	Range
Age	61.07	9.7	33–84
Education	15.48	3.1	5–26
% Male	65.1	–	–
% Caucasian	92.8	–	–
% African American	1.4	–	–
% Asian	1.9	–	–
% Other	3.9	–	–
Motor Severity	20.03	9.2	2–51
GDS-15	2.46	2.6	0–14
STAI-T	32.7	9.5	26–37
MOCA	27.14	2.32	–
Immediate Verbal Memory (z-score)	−0.17	0.94	–
Delayed Verbal Memory	−0.19	0.97	–
Visuospatial	0.01	0.96	–
Working Memory/Attention	−0.01	0.89	–
Verbal Fluency	−0.14	0.91	–
Processing Speed	−0.17	0.85	–
% Clinically Depressed at Baseline Visit**	16.0	–	–
% Clinically Depressed at any Visit**	35.9	–	–

**Clinical depression based on recommended > 5 cut-off for individuals with Parkinson’s disease [21]; GDS-15 = Geriatric Depression Scale-15 item; STAI-Trait = State-Trait Anxiety Inventory- Trait Subscale.

## 6 Multilevel models

**Fig. 1:**
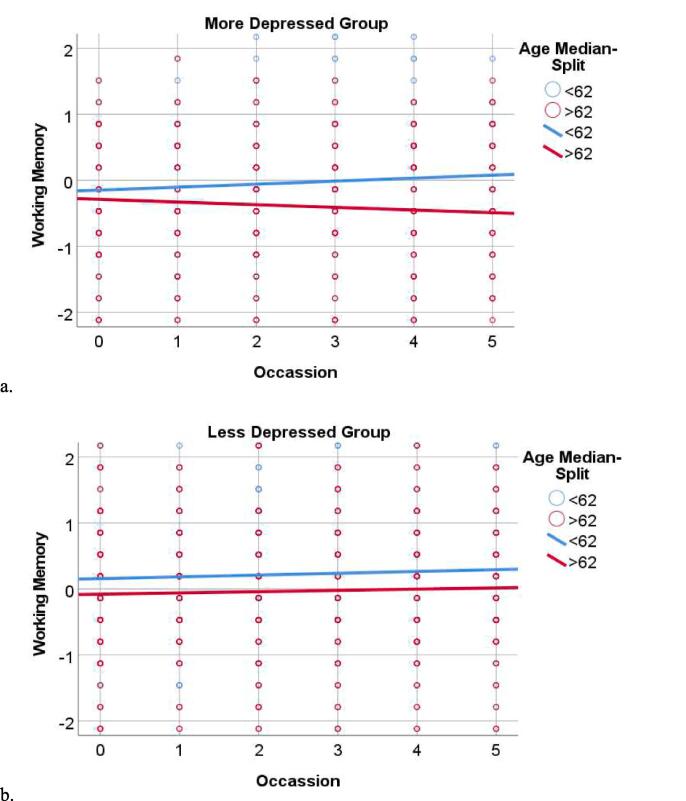
Age X Occasion X Depression Interaction and Working Memory. The figure depicts the longitudinal association between age and working memory among individuals with more depression (Fig. 1a) and less depression (Fig. 1b). The “more depressed” and “less depressed” groups were classified based on a median split (<3 vs ≥ 3); similarly the age groups were classified by a median split. Median splits were used for depiction purposes only, analyses treated age and depressive symptoms as continuous variables. Working memory scores (y axis) are depicted in a z-metric. Occasion (x-axis) represents the year of the study (i.e. baseline, first annual follow-up… 5th annual follow-up).

**Fig. 2:**
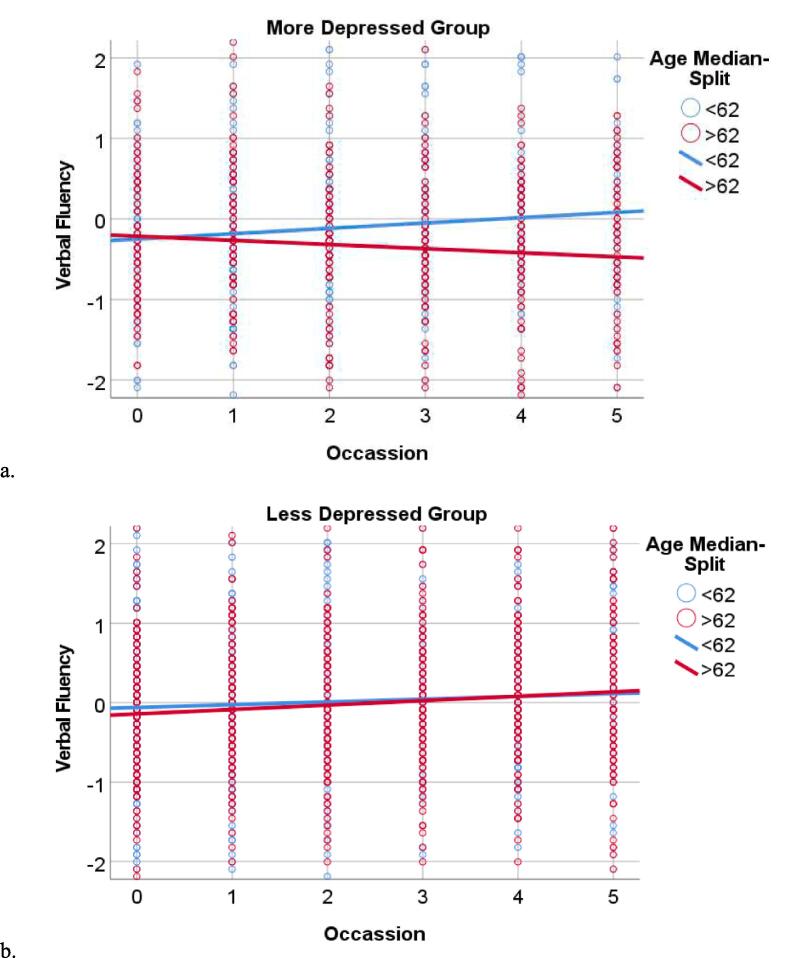
Age X Occasion X Depression Interaction and Verbal Fluency. The figure depicts the longitudinal association between age and verbal fluency among individuals with more depression (Fig. 1a) and less depression (Fig. 1b). The “more depressed” and “less depressed” groups were classified based on a median split (<3 vs ≥ 3); similarly the age groups were classified by a median split. Median splits were used for depiction purposes only, analyses treated age and depressive symptoms as continuous variables. Verbal fluency scores (y axis) are depicted in a z-metric. Occasion (x-axis) represents the year of the study (i.e. baseline, first annual follow-up… 5th annual follow-up).

Multilevel modeling was used to examine the longitudinal association between age, depressive symptoms, and cognitive functioning (Table 2). The Age X Occasion X Depressive symptoms interaction was significantly associated with tests of working memory/attention (Fig. 1) and verbal fluency (Fig. 2), but not the other cognitive tests. Specifically, the Age X Occasion X Depressive symptoms demonstrated that age-related longitudinal declines on both tests were more pronounced for individuals reporting more severe depressive symptoms; conversely, the negative impact of aging on cognition was less pronounced among individuals reporting less severe depressive symptoms. Working memory/attention was negatively associated with both age and the main effect of depressive symptoms.Verbal fluency was also negatively associated with motor severity and the main effect of depressive symptoms.

**Table 2:** Multi-level Modeling of Cognitive Domains.

Parameter	Verbal Fluency		Working Memory/ Attention		Processing Speed		Visuospatial Functioning		Immediate Memory		Delayed Memory		Global Cognition	
	B	p	B	p	B	p	B	p	B	p	B	p	B	p
Motor Severity	−0.079	**0.003**	0.002	0.931	−0.14	**<0.001**	−0.055	0.052	−0.072	**0.025**	−0.043	0.196	−0.100	**<0.001**
Age	0.031	0.407	−0.116	**0.003**	−0.069	0.069	−0.16	**<0.001**	−0.053	0.116	−0.074	**0.023**	−0.320	**<0.001**
Occasion	0.057	**<0.001**	−0.019	0.237	0.041	**0.011**	−0.011	0.511	0.104	**<0.001**	0.111	**<0.001**	−0.032	0.049
Depression	−0.064	**0.002**	−0.077	**<0.001**	−0.109	**<0.001**	−0.084	**<0.001**	−0.049	0.066	−0.022	0.398	−0.085	**<0.001**
Age X Occasion	−0.027	0.100	−0.027	0.106	−0.067	**<0.001**	−0.005	0.739	−0.029	0.152	−0.020	0.355	−0.070	**<0.001**
Age X Depression	−0.034	0.121	−0.009	0.738	−0.001	0.978	0.010	0.708	−0.026	0.339	0.001	0.975	−0.023	0.381
Occasion X Depression	−0.044	**0.014**	−0.019	0.246	−0.04	**0.022**	−0.009	0.601	0.012	0.610	−0.009	0.71	−0.067	**<0.001**
Age X Occasion X Depression	−0.051	**0.005**	−0.041	**0.036**	0.012	0.494	0.011	0.558	−0.006	0.801	0.009	0.734	−0.034	0.909

B = standardized estimates; significant values (p < 0.05) are bolded.

Although the three-way interaction was not associated with other cognitive domains, the Occasion X Depressive symptoms interaction term was significantly associated with processing speed and global cognition performance. This interaction term can be interpreted as individuals with more depressive symptoms experience greater longitudinal declines in processing speed and global cognition.

The main effect of depressive symptoms, but no interaction term involving depressive symptoms, was significantly associated with worse performance on visuospatial functioning. Lastly, performance on tests of immediate memory and delayed memory was not associated with any of the depressive symptoms variables.

## 7 Discussion

The purpose of this study was to examine the longitudinal association between depressive symptoms and cognitive decline as a function of age among patients with PD. We hypothesized that older PD patients with more severe depressive symptoms would be at greater risk of cognitive decline relative to their younger or less depressed counterparts. Our results supported this hypothesis, such that more severe depressive symptoms were associated with age-related declines in the domains of verbal fluency and working memory.

The current literature on the associations between depression and cognitive functioning among individuals with PD has resulted in some mixed findings, particularly when it comes to the role of depression in executive functioning. Two separate papers by Petkus and colleagues [[19], [31]] reported an association between executive functioning and depression. One cross-sectional study of 187 individuals with PD reported that worse executive functioning was associated with more depression, as well as anxiety and apathy [19]. In a second paper, lower cognitive scores across all domains, including aspects of executive functioning such as verbal fluency and working memory, were predictive of longitudinal worsening of depressive symptoms among the PPMI cohort [31]. However, other studies have failed to report significant associations between depression and tests of executive functioning [[11], [12], [13], [14]]. This may be due, in part, to differences in selection of executive function tasks across studies. Findings from the current study suggest that age moderates the association between depression and aspects of executive functioning (e.g. verbal fluency and working memory) but not other cognitive tasks. Indeed, studies from healthy aging populations suggest that executive functioning may be particularly sensitive to the interactive effects of aging and depression.

In adults without PD, depression is a risk factor for cognitive decline, and the risk increases with age [32]. In those with younger onset depression, cognitive changes are thought to occur as a result of longer durations of depressive episodes or increased number of lifetime episodes. This may contribute to disruptions in episodic memory [33], which increases with age and may be related to chronic stress (via HPA-axis disruption), and not necessarily due to Alzheimer-related neurodegenerative factors [34], though studies investigating these underlying mechanisms are still ongoing.

In contrast, depression that begins around or after the age of 60 year (i.e., late-onset depression) is hypothesized to be a contributor to, or result of, accelerated brain and cognitive aging and both major and minor depression in older adults increases the risk of cognitive decline and conversion to dementia [34]. This may be due to both depression and aging having combined adverse effects on similar neural networks [[35], [36], [37]], neurotransmitter functions [38], and other biological processes (e.g., vascular changes, neuroinflammation) [[39], [40]].

Due to these underlying mechanisms, late-life depression typically shows a fronto-subcortical pattern of deficits on formal cognitive tests (i.e., executive dysfunction, slowed processing speed) [41]. Interestingly, other research has shown different cognitive profiles emerge in cross-sectional studies of healthy older adults with depression (i.e., high performers, normal albeit lower performers, and low executive functions) [42], suggesting heterogeneity in cognitive functions in this population and specific subgroups that may be at increased risk for cognitive decline. Other studies have found that apathy, a common non-motor symptom in PD, mediates the relationship between executive function performance and depressive symptoms in older adults [43], without similar relationships seen in younger adults [44]. Apathy increases with age and is common in older adults with depression [45], highlighting another possible pathway through which geriatric depressive symptoms and PD may be related and affecting frontal-lobe mediated cognitive functions [42]. Moreover, there is evidence to suggest that late-onset depression may indicate a future diagnosis of PD or dementia with Lewy bodies [46], as those with late-onset depression are at greater risk of developing both motor and non-motor features and increased risk for abnormal dopamine transporter scans compared to healthy controls. This suggests that, in some individuals, late-onset depression may reflect a prodromal phase of PD.

Amongst some of the limitations of our study are the uneven representation of male and female participants, and predominantly Caucasian sample. In addition, depression was not ascertained via a structured clinical interview, but rather was determined via self-report on a subjective depression rating scale. Depression in the current PD sample was not prevalent. The sample of PD patients with depression at any visit was 35.9 % (175 participants out of 487 total) GDS-15 scores were low on average (mean = 2.5). Thus, the current findings may not generalize to a sample of clinically depressed PD patients. Similarly, the GDS-15 is a screening tool, rather than a diagnostic assessment tool. Future studies utilizing more rigorous assessment methods are needed to support our findings. Corrections for multiple comparisons were not conducted in this paper, therefore future studies are needed to replicate findings. Despite the limitations, our current work highlights the need for future studies investigating the effects of depression on cognitive decline in patients with PD. Additional work examining the underlying (or converging) mechanisms of depression in PD [47] are also needed to further understanding of these complex relationships.

In conclusion, the current study found that older PD individuals displayed more cognitive decline over time than younger and less depressed PD patients in the cognitive domains of language and working memory/attention. Older adults with PD may be more vulnerable to the neurotoxic effects of depressive symptoms (e.g., neuroinflammation, HPA axis disruption), which possibly contributes to these findings. Better monitoring/detection and management of depression in patients with PD via behavioral interventions (e.g., psychotherapy, cognitive remediation), exercise, and/or pharmacotherapy could potentially reduce cognitive decline and risk for conversion to dementia.

## CRediT authorship contribution statement

**Lea Hemphill:** Conceptualization, Formal analysis, Writing – original draft. **Yenny Valenzuela:** Conceptualization, Writing – original draft. **Kenya Luna:** Formal analysis, Writing – original draft. **Sarah M. Szymkowicz:** Supervision, Writing – review & editing. **Jacob D. Jones:** Conceptualization, Formal analysis, Supervision, Writing – review & editing.

## Declaration of Competing Interest

The authors declare that they have no known competing financial interests or personal relationships that could have appeared to influence the work reported in this paper.
